# Strain and serenity: exploring the interplay of stress, burnout, and well-being among healthcare professionals

**DOI:** 10.3389/fpsyg.2024.1415996

**Published:** 2024-06-25

**Authors:** Simona Dobešová Cakirpaloglu, Panajotis Cakirpaloglu, Ondřej Skopal, Barbora Kvapilová, Tereza Schovánková, Šárka Vévodová, Jane Peta Greaves, Alison Steven

**Affiliations:** ^1^Department of Humanities and Social Sciences, Faculty of Health Science, Palacký University Olomouc, Olomouc, Czechia; ^2^Department of Psychology, Faculty of Arts, Palacký University Olomouc, Olomouc, Czechia; ^3^Department of Psychology and Abnormal Psychology, Faculty of Education, Palacký University Olomouc, Olomouc, Czechia; ^4^Science and Research Centre, Faculty of Health Science, Palacký University Olomouc, Olomouc, Czechia; ^5^Department of Nursing, Midwifery and Health, Faculty of Health and Life Sciences, Northumbria University, Newcastle upon Tyne, United Kingdom

**Keywords:** stress, burnout, mental health, well-being, healthcare workers

## Abstract

**Introduction:**

Stress and burnout can negatively affect performance, mental health, and the overall well-being of healthcare workers. The study aims to examine the prevalence of stress and burnout, and investigate links between stress, burnout, mental state, and well-being among healthcare workers in the Czech Republic.

**Methods:**

A cross-sectional survey was conducted in the Czech Republic, focusing on healthcare professionals working in various healthcare settings. A total of 1,064 healthcare workers participated in the study. A standardized questionnaire battery was used, consisting of the Maslach Burnout Inventory (MBI), Perceived Stress Scale (PSS) and Supso-7 measuring mental state. Separate correlation and multiple regression analyses were conducted.

**Results:**

46.24% of the healthcare workers reported high levels of emotional exhaustion, 25.56% reported high levels of depersonalization, 24.15% reported low levels of personal accomplishment, while 11.18% reported high levels of perceived stress. The findings revealed that emotional exhaustion, a core component of burnout, was associated with increased feelings of anxiety and depression. Perceived stress was also linked to anxiety and depression, while personal accomplishment appeared to mitigate depression and support positive psychological well-being.

**Conclusion:**

The study provides promising evidence suggesting that addressing stress and emotional exhaustion, while fostering a sense of personal achievement, could lead to improvements in the mental health and work performance of healthcare workers. These findings highlight the importance of addressing burnout and stress management strategies to support the overall well-being of healthcare professionals.

## 1 Introduction

In the Czech Republic, healthcare professionals operate within a system that combines both public and private healthcare services. The country has a universal healthcare system funded through public health insurance contributions, providing residents with access to essential medical care. Healthcare professionals in the Czech Republic, including doctors, nurses, and specialists, face challenges typical of many European healthcare systems, such as increasing demands due to aging populations and the need for modernization of facilities and technologies within the healthcare system. Shortage of staff can lead to increased workloads, longer hours, and heightened stress levels among employees. Demanding conditions and high-pressure environments contribute to various psychosocial risks, including burnout. The concept of burnout syndrome was first used by Herbert J. Freudenberg in 1974, when he observed a loss of motivation and a decreased readiness to meet work commitments and stay on the job among volunteers in a psychiatric clinic. Christine Maslach, who developed the famous Maslach Burnout Inventory (MBI), describes the problem as a “prolonged response to chronic interpersonal stressors on the job. The three key dimensions of this response are overwhelming exhaustion; feelings of cynicism and detachment from the job; and a sense of ineffectiveness and failure”(Maslach, 1998). Maslach suggests that burnout can negatively affect personal and social functioning. While some individuals may quit their job due to burnout, others may continue working but with reduced productivity (Maslach, 1998). These reductions in work quality and physical and mental health may be costly, not only to the individual but also to the team and the organization (Koren et al., 2023).

Maslach and Leiter (2016) describe the cause of burnout syndrome as a long-term mismatch between an individual and at least one of the six dimensions of the work process which they identify as workload, control, reward, community, fairness, and values. Work-overload can contribute to burnout by depleting an individual’s ability to meet job demands, particularly when it is prolonged and there are no opportunities for rest, recovery, and rebalancing. Lack of control over the work process can reduce motivation, performance and lead to stress and burnout. A lack of financial, institutional, or social rewards can be interpreted as a reduced self-efficacy and inferior performance, leading to frustration, a reduced self-concept, decreased work motivation, and an increased susceptibility to burnout. Lack of support and trust in social relationships, as well as unresolved conflicts, can increase the risk of burnout. Reactions to lack of recognition and perceived unfairness, such as anger, cynicism, and hostility, can limit the individual’s ability to perform their job and lead to burnout (Maslach and Leiter, 2016; Dall’Ora et al., 2020; Taranu et al., 2022; Luna et al., 2023).

Conflict between individual and organizational values can often force employees to act in ways that are inconsistent with their roles or qualifications, leading to burnout (Moss, 2021; Rotenstein et al., 2023). Burnout’s depletion of mental or physical energy is associated with prolonged job stress and an individual’s inability to choose effective coping strategies (Edward and Hercelinskyj, 2007). Burnout is a major problem, commonly associated with helping professions, especially in healthcare (Hellesøy et al., 2000) but it may also occur in many other sectors (Scanlan and Still, 2013; Makara-Studzińska et al., 2021).

A meta-analysis by Rotenstein et al. (2018) analyzed various studies on burnout syndrome in physicians between 1991 and 2018, which included over 109,000 physicians from 45 countries. The findings confirmed a significant association between burnout syndrome higher rates of medical errors and reduced physician work performance, which led to lower patient satisfaction with physician attitudes and prolonged patient recovery (Shanafelt et al., 2016; Rotenstein et al., 2018). In the meta-analysis of various studies conducted by Molina-Praena et al. (2018), shift work, multiple jobs, lack of recognition and appreciation for work well done, and length of experience were identified as factors contributing to burnout syndrome among nurses. Higher levels of job satisfaction and fulfilment among nurses have been associated to two factors: feeling that their work has a positive impact on patients and having enough time to devote to their work (Molina-Praena et al., 2018). Excessive administrative workload reduces nurses’ time with patients and can increase the risk of development of burnout syndrome.

### 1.1 The prevalence of burnout among healthcare professionals

Burnout syndrome has been reported to have increased more among healthcare professionals than in any other profession Healthcare workers not only face physical demands and pressures related to patient care, but they are constantly exposed to emotional challenges associated with illness, human suffering, death, and daily stressful situations heightened by major events such as the pandemic, wars etc. (Bridgeman et al., 2018; Ghahramani et al., 2021; Macaron et al., 2023).

Discrepancies between assessments of the prevalence of burnout can be explained by several conceptual, methodological, socio-demographic, occupational and other factors. Back in 2007 Embriaco et al. (2007) revealed an alarming rate of severe burnout syndrome among healthcare workers. According to the study, approximately 50% of physicians and one-third of critical care nurses suffer from burnout. Shanafelt et al. (2012) revealed that 45.8% of 7,288 U.S. physicians had at least one burnout symptom. Besides the overall score, researchers also examined the prevalence of specific burnout components. More recently a cross profession review covering 182 studies from 45 countries indicated significant differences in the estimates of burnout syndrome between general practitioners and nurses (Rotenstein et al., 2018). For instance, the prevalence rates in U.S. ranged from 9.8–45%, 12.1–69% in China, 7.3% in Spain while studies conducted in U.K. reported a 19.8% prevalence rate.

In relation to single profession the trend continues with finding that 11.23% of nurses worldwide experienced burnout symptoms, based on findings of 113 studies in a systematic review and 61 studies in a meta-analysis (Woo et al., 2020). For instance, in a meta-analysis of studies of medical nurses conducted by Molina-Praena et al. (2018), 31% suffered from emotional exhaustion (EE), 24% from depersonalization (DP), and 38% from low personal achievement. In the United Kingdom, physicians scored from 31 to 54.3% for emotional exhaustion, from 17.4 to 44.5% for depersonalization, and from 6 to 39.6% for low personal accomplishment (Imo, 2017). A recent Greek study by Konstantinou et al. (2018) had similar findings, reporting 53.8% of mental health nurses experiencing high levels of emotional exhaustion (EE), 24.4% high levels of depersonalization (DP) and 25.6% high levels of personal accomplishment (PA). A survey of healthcare workers in Czech and Slovak university hospitals conducted between 2021 and 2022 revealed that emotional exhaustion (EE) was found in 53.2% of respondents; depersonalization (DP) in 33% of them, and low personal accomplishment (PA) in 47.8% of them (Štěpánek et al., 2023).

However, it is not only qualified professionals that are at risk of burnout, stress, and emotional exhaustion, with studies identifying significant levels of these states in students (Morales-Rodríguez et al., 2019; Valero-Chillerón et al., 2019; Potter and Cadizet, 2021). Jezzini-Martinez et al. (2023) conducted a study at a medical school in Mexico which found a high prevalence of burnout syndrome even among medical students. According to the study, 54.2% of students had symptoms of burnout, including high levels of emotional exhaustion (79.6%), cynical feelings (57.3%) and low academic effectiveness (36.4%).

### 1.2 Burnout relation to mental-health and well-being

While there exists a certain degree of variability in research findings regarding the prevalence rates of burnout among physicians and nurses, there is a consensus within the scientific community that this phenomenon is escalating and exerting substantial repercussions on the physical and mental well-being of individuals in healthcare professions. Notably, burnout syndrome frequently coexists with post-traumatic stress disorder (PTSD), anxiety, depersonalization, heightened emotional exhaustion, reduced subjective well-being, and other psychopathological conditions. A study by Colville et al. (2017) revealed that 13% of participants exhibited clinically significant posttraumatic stress symptoms, emphasizing a correlation between burnout and various stress-related indicators, particularly anxiety.

Studies related to the stress of healthcare environments for professions have shown that physicians and nurses consistently face excessive pressure from work overload and responsibility for the health and lives of others, causing chronic stress (Rotenstein et al., 2018). Extensive evidence supports the notion that stress and burnout can be linked to elevated levels of anxiety, depression, and a general decline in well-being across multiple professional domains, encompassing both the broader workforce and healthcare professions. A study involving 645 U.S. general surgery residents conducted by Smeds et al. (2020) uncovered associations between perceived stress, burnout, and depression. Furthermore, observations by Hou et al. (2022) during the COVID-19 pandemic highlighted that work-related stress predicted heightened anxiety levels among 798 Chinese medical workers.

The long-term ramifications of chronic stress and burnout extend beyond immediate consequences, involving the depletion of physical, emotional, and psychological energy resources. Such depletion sets off a cascade of losses that may extend to other dimensions, including a decline in feelings of self-efficacy and the adoption of maladaptive coping strategies (Maddock, 2024). Consequently, it is reasonable to assert that chronic stress and burnout contribute significantly to adverse mental health effects, including anxiety, depression, and overall diminished well-being, thereby impacting various facets of an individual’s life (Hakanen and Schaufeli, 2012). Hobfoll’s resource theory explains this relationship. According to this theory, individuals have limited personal and environmental resources (like physical, psychological, and social resources) to manage stress and challenges. Chronic stress and burnout deplete these resources, making individuals more susceptible to mental health issues.

In healthcare, professionals face high demands such as long hours, heavy patient loads, and emotional strain, leading to chronic stress and burnout. Coping with these demands depletes personal and environmental resources, resulting in emotional exhaustion, depersonalization, and reduced personal accomplishment—common signs of burnout (Hobfoll, 1989). This underscores the enduring spill-over effects of burnout on depression, emphasizing the importance of supportive interventions within the work environment of healthcare professionals. The novelty of this article lies in its investigation of stress, burnout, and mental well-being specifically among healthcare workers in the Czech Republic, providing new insights into the prevalence and impact of these factors in this context. Focusing on specific occupational groups within healthcare, such as nurses, physicians, and midwives, is quite common in research on stress and burnout. However, it seems that there’s a gap in the Czech Republic regarding studies that look at healthcare professionals as a whole, rather than individually. This broader perspective could provide valuable insights into systemic issues affecting the entire healthcare workforce and might lead to more comprehensive interventions and support mechanisms.

The aim of this study is to provide a greater understanding of the potential relationships between stress, burnout (emotion exhaustion, depersonalization, and personal accomplishment), mental state and well-being in healthcare workers. To achieve this aim, this study has two objectives:

1. To identify the rates of stress, burnout, mental state, and well-being of healthcare workers from a sample of healthcare workers in the Czech Republic.
2. To examine the relationships between stress, burnout (emotion exhaustion, depersonalization, and personal accomplishment), mental state and well-being in a group of healthcare workers in the Czech Republic.

The following Research Question was formulated: What is the effect of stress and burnout on the psychological well-being and mental state of healthcare workers?

## 2 Methods

### 2.1 Participants and procedure

The present study is a cross-sectional survey of healthcare employees in the Czech Republic. Data was collected electronically using Google forms, which met the methodological and research criteria of online research relevance (i.e., high degree of security, archiving and encoding during data transfer, access via generated password). The link to the survey, was distributed via email to healthcare institutions in all regions of the Czech Republic, in both public and private sectors. This direct approach to health care organizations was used to mitigate against false, imposter and, or ‘bot’ generated submissions. Data collection took place between September 2023 and November 2023. A total of 1,064 healthcare workers participated in the study working in various healthcare settings. The characteristics of the participants are shown in Table 1.

**Table 1:** Characteristic of research sample.

**Characteristic of research sample**		**No. (%)** **(*n* = 1,064)**
**Sex**		
	Male	251 (23.59)
	Female	813 (76.41)
**Education level – no. (%)**		
	Secondary vocational edu w/o exam	13 (1.22)
	Secondary education w/exam	183 (17.2)
	Higher specialized education	49 (4.61)
	College undergraduate degree – BS, BA.	122 (11.47)
	College graduate degree – Master’s degree.	404 (37.97)
	College postgraduate degree – PhD degree	293 (27.54)
**Job position – no. (%)**		
	Physician	457 (42.95)
	Non-medical healthcare worker	585 (54.98)
	Other	22 (2.07)
**Sector – no. (%)**		
	State	447 (42.01)
	Private	617 (57.99)
**Work schedule**		
	A dayshift	722 (67.86)
	2-shift regimen	245 (23.03)
	3-shift regimen	97 (9.12)
**Lead worker**		
	Yes	550 (51.69)
	No	514 (48.31)
**Characteristic**		**Mean ± SD**
Age – yr		46.58 ± 11.97
Length of employment – yr		21.94 ± 11.43
Length of employment at current workplace – yr		13.01 ± 10.32

### 2.2 Ethical consideration

All participants were assured of the confidentiality of their answers and signed an online informed consent form prior to undertaking the questionnaire. No specific information enabling the identification of specific respondents was obtained as part of the online data collection. The Research Ethics Committee of Health Sciences, Palacký University Olomouc (UPOL-18671/FZV2023) granted ethical approval for this study. The inclusion criteria for this study were: being a healthcare worker in the Czech Republic working in the private or public sector with a minimum of 1-year length of practice. All participants received detailed written information about what participation in the study would entail, and were provided with several opportunities to ask questions before they provided written consent.

### 2.3 Measures

The participants were asked to provide demographic information and to fill in self-report measures. The reliabilities of each self-report measure were calculated based on the responses given by the participants.

The Perceived Stress Scale (PSS: Cohen et al., 1983): The PSS is a ten-item, five-point Likert scale (0 = never; 4 = very often), measure of perceived stress, which is widely used in the research literature on stress (Cohen et al., 1983). Some sample items related about data collection instrument are the following: “*In the last month, how often have you been upset because of something that happened unexpectedly*?”; “*In the last month, how often have you felt nervous and “stressed*”? Higher perceived stress is indicated by higher scores. The reliability of the PSS in this study was deemed acceptable (Cronbach’s *α* = 0.87).

The Maslach Burnout Inventory (MBI: Maslach et al., 1997): The MBI is the most widely used occupational burnout measure. It is a valid and reliable measure, with its discriminant and convergent validity confirmed with a range of populations (Maslach et al., 1996). The MBI has 22 items, each scored on a seven-point Likert scale (0 = never; 6 = everyday) and contains three subscales measuring emotional exhaustion (MBI-EE), depersonalization/loss of empathy (MBI-DP) and personal accomplishment (MBI-PA). The examples of the sample items for each subscale are the following: “*I feel emotionally drained by my work*” (MBI-EE); “*I really do not care about what happens to some of my patients/clients*” (MBI-DP); “*I feel full of energy*” (MBI-PA). The Cronbach’s alphas for emotional exhaustion, depersonalization and personal accomplishment in this study were 0.92, 0.74, and 0.75, respectively.

The SUPSO-7 test is used to measure changes in the current psychological state depending on influencing situational variables. It allows for the understanding and interpretation of the relationships between internal and external manifestations of an individual, classifying situational variables from the perspective of optimal (stimulating psychological development) and suboptimal (leading to maladaptive manifestations or psychological distress). The method enables the assessment of the usual, long-term, and updated psychological state resulting from the influence of various situational factors. The SUPSO-7 test consists of 7 subscales specifically designed to measure participants both mental state and psychological well-being. The psychological well-being is measured by the scale P (well-being). SUPSO-7 is the result of a factor analysis of operationally defined and pragmatically designed scales comprising 28 adjectives (Mikšík, 2005). The test covers the following subscales: D (depression), U (anxiety), S (sadness), O (impulsivity), A (activity), N (mental restlessness), and P (well-being). The participant on a 4-point scale has to indicate his usual experiences and states. The examples of the sample items are the following: Depression (*pessimistic*); Anxiety (*tense*); Well-being (*content*); Activity (*energetic*); Impulsivity (*explosive*); mental restlessness (*impatient*); Sadness (*unhappy*). The Cronbach’s alpha coefficient is α = 0.86 for the 28 items.

Sociodemographic questionnaire is focused on sociodemographic data such as age, gender, length of employment, length of employment at current workplace, job position, work schedule, sector of employment, the highest completed level of education, place of work (region, size of the municipality).

### 2.4 Data processing and evaluation

In the first stage, the data were transformed into an xlsx format compatible with MS Excel 2013, which can easily process data exported from the electronic questionnaire. The research study was designed as a quantitative survey. Data collection was performed electronically using Google Forms, which met the methodological and research criteria of online research relevance. During the second stage, the data were formally and logically inspected. No missing values were detected. Data integrity was ensured during the data collection, which did not allow any missing data. For the scales of the SUPSO-7 questionnaire, gender-specific weighted scores were first calculated according to the method manual. Further data processing was performed using the STATISTICA program, version 13.

An analysis of results distribution confirmed normal data distribution; for this reason, a parametric statistical approach was selected. Correlation and regression analyses were performed rather than structural equation modelling due to: (1) the aims of the study and (2) the fact that this study is somewhat exploratory in nature, as the potential predictors and outcomes being tested were generated from a review of the literature, rather than from a theoretical framework. We screened the data for missing values and potential outliers, employing the interquartile rule as described by Hoaglin et al. (1986), utilizing whisker plots for visualization. No outliers were found. Separate correlation and then multiple regression analyses were conducted using perceived stress, emotional exhaustion, depersonalization and personal accomplishment as predictor variables and the variables of the SUPSO questionnaire as outcomes. All regression assumptions were met. The scatterplot diagrams showed that there were linear relationships between each predictor variable and the outcome. We used the Durbin–Watson statistic to check if the residuals were independent. A value of around two on the Durbin–Watson scale (which ranges from zero to four) suggests that there is little to no correlation among residuals (Durbin and Watson, 1992). There was independence of residuals, as demonstrated by a Durbin–Watson statistic of 1, 92 for the regression on P (well-being), 1.98 for the regression on A (activity), 2.00 for the regression on O (impulsivity), 2.07 for the regression on N (mental restlessness), 1.98 for the regression on U (anxiety), 1.93 for the regression on D (depression) and 1.98 for the regression on S (sadness). We visually examined the plot of standardized residuals against standardized predicted values to evaluate homoscedasticity. Additionally, a normal probability plot indicated that these residuals followed a normal distribution. Compliance with the conditions for the use of regression analysis was verified prior to data analysis. The tests were conducted at 5% level of significance.

## 3 Results

The first part presents basic findings concerning the numbers and proportions of burnout among healthcare workers. Table 2 presents the proportions of the 3 subscales of the MBI questionnaire. The proportion of healthcare workers reporting low-level emotional exhaustion was 235 30.55% with 23.21% reporting moderate levels of emotional exhaustion and 46.24% reporting high levels of emotional exhaustion. The proportion of healthcare workers reporting low-level depersonalization was 45.02% per cent, with 29.42% reporting moderate levels of depersonalization and 25.56% reporting high levels of depersonalization. The proportion of healthcare workers reporting low-level personal accomplishment was 24.15%, with 33.83% reporting moderate levels of personal accomplishment and 42.01% reporting high levels of personal accomplishment. The proportion of healthcare workers reporting low-level of perceived stress was 37%, moderate level 52% while 11% reported high-level stress.

**Table 2:** The proportions of burnout and stress among healthcare workers.

	**Emotional Exhaustion** **no. (%)**	**Depersonalisation** **no. (%)**	**Personal Accomplishment** **no. (%)**	**Stress** **no. (%)**
Low	235 (30.55)	479 (45.02)	257 (24.15)	390 (36.65)
Moderate	247 (23.21)	313 (29.42)	360 (33.83)	555 (52.16)
High	492 (46.24)	272 (25.56)	447 (42.01)	119 (11.18)

From Table 3, it is evident that high scores in the Perceived stress scale measure along with the scales of emotional exhaustion and depersonalization are most likely contributing to increased feelings of sadness, anxiety, and depression among the targeted employees, as well as an overall decline in mental well-being and activity. The first scale, emotional exhaustion, shows significantly higher values in the mid-range correlation of the SUPSO questionnaire, particularly in relation to depression (*r* = 0.76), sadness (*r* = 0.60), and anxiety (*r* = 0.59). The deterioration of mental well-being associated with emotional exhaustion is also confirmed by negative correlation values on the mental well-being scale (*r* = −0.65) and activity (*r* = −0.46).

**Table 3:** Means, standard deviation and Pearson correlations of total scales (*n* = 1,064).

	***M***	**SD**	**Emotional Exhaustion**	**Depersonalization**	**Personal accomplishment**	**Stress**
A-Active	6.22	2.37	−0.46**	−0.29**	0.52**	−0.40**
O-Impulsivity	2.73	2.28	0.34**	0.42**	−0.34**	0.37**
N-Mental restless	3.65	2.29	0.57**	0.46**	−0.39**	0.54**
D-Depression	3.89	2.46	0.76**	0.46**	−0.41**	0.59**
U-Anxiety	3.76	2.64	0.59**	0.36**	−0.37**	0.63**
S-Sadness	3.59	2.16	0.60**	0.40**	−0.43**	0.61**
P-Mental well-being	7.11	2.16	−0.65**	−0.43**	0.58**	−0.59**

**p < 0.01, *p < 0.05.

The second scale of the MBI, depersonalization, also exhibits significantly higher values in the mid-range correlation of the SUPSO questionnaire, particularly in relation to depression (*r* = 0.46), mental restlessness (*r* = 0.46), and sadness (*r* = 0.40). The decline in mental well-being associated with this factor of burnout is further confirmed by negative correlation values on the mental well-being scale (*r* = −0.43) and activity (*r* = −0.29).

The third scale of the MBI, personal accomplishment, appears to be a protective factor against depression and in favor of positive mental well-being. It also exhibits significantly higher values in the mid-range correlation of the SUPSO questionnaire, particularly in relation to depression (*r* = −0.41), mental restless (*r* = −0.39), anxiety (*r* = −0.37), and sadness (*r* = −0.43). The increase in mental well-being associated with this factor of burnout is further confirmed by positive correlation values on the mental well-being scale (*r* = 0.58) and activity (*r* = 0.52).

The perceived stress scale PSS shows significantly higher values in the mid-range correlation of the SUPSO questionnaire, particularly in relation to depression (*r* = 0.59), sadness (*r* = 0.61), and anxiety (*r* = 0.63). The deterioration of mental well-being associated with perceived stress is also confirmed by negative correlation values on the mental well-being scale (*r* = −0.59) and activity (*r* = −0.40).

Other results relate to answering the research question. Primarily, this study investigated whether stress and burnout affect well-being and mental state of the healthcare employees.

The results of the regression analysis for the dependent variables “D (depression),” “U (anxiety),” “S (sadness),” “O (impulsivity),” “A (activity),” “N (mental restlessness),” and “P (well-being)” indicate high statistical significance and strong explanatory power of the models. The models explain variability in the dependent variables ranging from 24 to 63%, as measured by the coefficients of determination (R2). The results showed that in the context of the possible influence of stress and burnout on mental state, the effect of emotional exhaustion, depersonalization, personal accomplishment, and perceived stress can be observed (Table 4). Emotional exhaustion had the strongest effect on depression (explaining 63% of its variance, *p* < 0.001), followed by the effect on sadness (48% of its explained variance, p < 0.001), anxiety (explaining 43% of its variance, *p* < 0.001). Depersonalization had the strongest effect on impulsivity (explaining 24% of its variance, *p* < 0.001). Personal accomplishment had the strongest effect on mental well-being (explaining 57% of its variance, *p* < 0.001) and activity (explaining 35% of its variance, *p* < 0.001). Perceived stress had the strongest effect on sadness (explaining 48% of its variance, *p* < 0.001).

**Table 4:** Linear regression of different psychological states (DV) as a function of different forms of burnout and stress (predictors).

**DV/Predictors**	***F* (df)**	***P***	**Adj. *R*** ^**2**^	**β**	***t***	**P**
Depression (D)	450.76 (4,1059)	*p* < 0.01	0.62			
*EE* *DP* PA PSS				0.553 0.044 −0.076 0.251	21.414 1.937 −3.510 10.370	0.000 0.052 0.000 0.000
Anxiety (U)	208.68 (4,1059)	*p* < 0.01	0.43			
*EE* *DP* PA PSS				0.339 0.029 −0.091 0.333	10.679 1.037 −3.448 11.182	0.000 0.299 0.000 0.000
Sadness (S)	247.34 (4,1059)	*p* < 0.01	0.48			
*EE* *DP* PA PSS				0.312 0.053 −0.144 0.344	10.230 1.985 −5.623 12.034	0.000 0.047 0.000 0.000
Mental restless (N)	196.93 (4,1059)	*p* < 0.01	0.42			
*EE* *DP* PA PSS				0.278 0.176 −0.094 0.274	8.645 6.212 −3.508 9.083	0.000 0.000 0.000 0.000
Impulsivity (O)	85.84 (4,1059)	*p* < 0.01	0.24			
*EE* *DP* PA PSS				0.010 0.285 −0.123 0.216	0.272 8.732 −3.996 6.242	0.785 0.00 0.00 0.00
Active (A)	147.08 (4,1059)	*p* < 0.01	0.35			
*EE* *DP* PA PSS				−0.29 0.06 0.40 −0.07	−8.622 2.090 14.026 −2.482	0.000 0.036 0.000 0.013
Mental well-being (P)	359.71 (4,1059)	*p* < 0.01	0.57			
*EE* *DP* PA PSS				−0.388 −0.005 0.342 −0.210	−14.024 −0.235 14.748 −8.108	0.000 0.813 0.000 0.000

EE, emotional exhaustion; DP, depersonalization; PA, personal accomplishment; PSS, perceived stress scale.

## 4 Discussion

The objective of this study was to examine the prevalence of burnout syndrome among healthcare professionals in the Czech Republic and to explore the association between stress, different dimensions of burnout and employees’ psychological well-being. The concept of burnout, introduced by Freudenberg in 1974 and further developed by Maslach, is clearly reflected in the reported dimensions of overwhelming exhaustion, cynicism, detachment, and a sense of ineffectiveness (Maslach, 1998). The interpretations of the findings align with the core dimensions of the Maslach Burnout Inventory (MBI), emphasizing emotional exhaustion, depersonalization, and reduced personal accomplishment. The study found that 30.55% healthcare workers reported low-level of emotional exhaustion, 23.21% reported moderate levels of emotional exhaustion and 46.24% reported high levels of emotional exhaustion. 45.02% of healthcare workers reported low-level of depersonalization, 29.42% reported moderate levels of depersonalization and 25.56% reported high levels of depersonalization. The proportion of healthcare workers who reported low-level of personal accomplishment was 24.15%, with 33.83% reporting moderate levels of personal accomplishment and 42.01% reporting high levels. The results also revealed that 37% of healthcare workers reported low-level of perceived stress, moderate level 52% while 11% reported high-level stress. Our findings align with those of various studies (Imo, 2017; Molina-Praena et al., 2018). Similarly, a recent study in Greece by Konstantinou et al. (2018) reported comparable findings. They found that 53.8% of mental health nurses experienced high levels of emotional exhaustion (EE), 24.4% dealt with high levels of depersonalization (DP), and 25.6% struggled with high levels of personal accomplishment (PA). The prevalence rates, as indicated by the responses of healthcare workers, underscore the pervasive nature of burnout in this specific context. The reported rates of emotional exhaustion among healthcare professionals point to the taxing nature of their work, often involving intense emotional labor and exposure to distressing situations (Gray, 2009; Qiu et al., 2023). The sense of depersonalization reflects a detachment from their roles, possibly as a coping mechanism in response to the challenging interpersonal aspects of healthcare work (Wang et al., 2020). Additionally, the diminished sense of personal accomplishment raises concerns about overall job satisfaction and fulfilment among healthcare professionals in the Czech Republic (Vňuková et al., 2023). In the context of Maslach and Leiter’s (2016) six dimensions of the work process, the interpretations suggest a potential misalignment between healthcare professionals and critical aspects such as workload, control, reward, community, fairness, and values. The demanding workload, coupled with a lack of adequate support and recognition, may contribute to the reported burnout levels. The study’s interpretations underscore the urgent need to address these dimensions systematically to create a work environment conducive to the well-being of healthcare professionals.

The other goal of the study is to investigate potential associations and effect between stress, different dimensions of burnout syndrome and the mental well-being of the individuals affected. The findings suggest that emotional exhaustion is a widespread risk factor for developing feelings of anxiety, depression, and mental well-being issues in healthcare workers. Moreover, perceived stress seems to contribute to anxiety and depression, while personal accomplishment appears to protect against depression and supports positive mental well-being. The findings of this study suggest that emotional exhaustion, depersonalization, and personal accomplishment have significant implications for the anxiety, depression, and overall psychological well-being of healthcare workers. There is abundant evidence suggesting a strong connection between stress, burnout, and increased levels of anxiety, depression, and overall decline in well-being across various professional sectors, including both the general workforce and healthcare fields (Çelmeçe and Menekay, 2020; Maddock, 2024). For example, a study conducted by Smeds et al. (2020) involving 645 US general surgery residents revealed correlations between perceived stress, burnout, and depression. Additionally, observations made by Hou et al. (2022) during the COVID-19 pandemic emphasized that work-related stress was a predictor of elevated anxiety levels among 798 Chinese medical workers. Another study involving 386 Korean nurses (Choi et al., 2018) revealed a correlation between emotional exhaustion and both anxiety and depression. Similarly, Koutsimani et al. (2019), in a systematic review and meta-analysis of 101 studies, discovered a significant link between emotional exhaustion and anxiety as well as depression. Additionally, personal accomplishment was found to be correlated with the subjective and psychological well-being of 274 German hospital physicians (Huber et al., 2020), as well as the psychological well-being of 216 US healthcare employees (Shuck and Reio, 2014). These findings shed light on the growing problem of burnout among healthcare professionals in the Czech Republic.

The implications of this study extend beyond the individual experiences of healthcare professionals to broader consequences for the healthcare system and the well-being of patients. The high prevalence of burnout among healthcare workers carries significant ramifications for the quality of patient care, and patient safety as burnout has been linked to medical errors and reduced patient satisfaction (Rotenstein et al., 2018). The findings underscore the urgency of implementing targeted interventions to mitigate burnout and enhance well-being of healthcare professionals. Organizational strategies aimed at managing workload, providing adequate support, and recognizing the contributions of healthcare workers are crucial. The study’s implications align with Maslach and Leiter’s (2016) emphasis on addressing the root causes of burnout by fostering a positive work environment. At the systemic level, the study’s results matter for healthcare policymakers and administrators. Burnout can lead to workforce shortages, reduced productivity, and increased healthcare costs (Rotenstein et al., 2018). Therefore, investing in interventions to prevent and alleviate burnout is not only a matter of prioritizing the well-being of healthcare professionals but also a strategic imperative for the overall functioning of the healthcare system in the Czech Republic. Interventions are needed at multiple levels- not only a focus on the individual as this can implicitly seem like ‘victim blaming’ as the individualization almost seems to suggest it is their problem for not being resilient or knowing how to deal with things- therefore interventions also need to be at organizational, ward or team levels. The implications also extend to the societal level, as burnout among healthcare professionals can have cascading effects on public health. A workforce experiencing burnout may be less resilient in the face of health crises, potentially compromising the overall health and well-being of the population. Thus, the study’s results emphasize the broader societal implications of addressing burnout in the healthcare sector.

## 5 Limitations

While the study provides valuable insights, it is crucial to acknowledge its limitations to avoid overgeneralization and misinterpretation of the findings. In opting to categorize all healthcare professionals together as a unified group in our study, we acknowledge the potential variations in organizational structures, work cultures, and professional autonomy across different healthcare systems. While our focus solely on health workers simplifies data collection and analysis, it’s essential to recognize that nuances in job roles and responsibilities could influence our findings. For instance, in the Czech healthcare system, where individuals may be expected to function as independent clinicians rather than solely following protocols, our results may bear unique implications. This approach allows for a more streamlined examination of broader trends and patterns within the healthcare workforce, facilitating a deeper understanding of professional dynamics and informing targeted interventions to enhance overall quality of care. The cross-sectional nature of the research design is a significant limitation, preventing the establishment of causal relationships. The reported burnout rates offer a snapshot, but the temporal dynamics and causation remain unclear. Future research employing longitudinal designs would be instrumental in unravelling the complex trajectories of burnout among healthcare professionals in the Czech Republic. The reliance on self-report measures introduces the possibility of response bias. Healthcare professionals may provide responses influenced by social desirability or may not accurately represent their experiences. Combining self-report measures with more objective indicators or employing a mixed-methods approach could enhance the validity of findings. This approach allows for triangulation of data from multiple sources, thereby reducing the impact of response bias and increasing the reliability of the study’s conclusions. The study’s focus on healthcare professionals in the Czech Republic may limit the generalizability of the findings to other cultural and organizational contexts. Cultural variations in work-related attitudes and behaviors should be considered in future research to understand how burnout manifests in different settings. Comparative studies across cultures could provide valuable insights into the cultural nuances influencing burnout among healthcare professionals. Moreover, the study primarily utilizes quantitative data, providing prevalence rates for burnout dimensions. While this offers a quantitative understanding, it may not capture the nuanced qualitative aspects of healthcare professionals’ experiences. Future research could explore the qualitative dimensions of burnout, employing methods such as interviews or focus group discussions to gain a deeper understanding of the contextual factors contributing to burnout as well as the effect of stress, burnout on the individuals’ wellbeing.

## 6 Conclusion

Building on the study’s contributions, recommendations for further research encompass several dimensions. Longitudinal studies are imperative to track the evolution of burnout among healthcare professionals over time. Understanding the antecedents and consequences of burnout longitudinally can provide a more nuanced understanding of the factors contributing to its development and persistence. Connecting with the global literature on burnout, future research could explore the effectiveness of organizational-level interventions in mitigating burnout among healthcare professionals in the Czech Republic. Strategies such as workload management, mentorship programs, and organizational support structures should be investigated to determine their impact on reducing burnout and enhancing overall well-being. By empirically evaluating the effectiveness of these interventions within the Czech healthcare context, researchers can provide valuable insights into strategies that best promote resilience and mitigate burnout among healthcare professionals. A cultural perspective should be integrated into future research to explore the unique cultural factors influencing burnout among healthcare professionals in the Czech Republic. Considering the potential impact of cultural dimensions on work-related attitudes and behaviors, cross-cultural studies could shed light on the cultural nuances contributing to burnout in this specific context. Moreover, the study highlights the need for a more comprehensive understanding of the qualitative aspects of burnout. Future research could employ mixed methods approaches, combining quantitative data with qualitative insights from healthcare professionals. In-depth interviews or focus group discussions could provide a richer understanding of the contextual factors and experiences that contribute to burnout. In conclusion, this study significantly contributes to the understanding of burnout among healthcare professionals in the Czech Republic, emphasizing the prevalence rates and implications for mental health. The interpretations align with established theories of burnout, and the implications underscore the urgency for targeted interventions. However, the study’s limitations necessitate future research endeavors, including longitudinal studies, cross-cultural investigations, and a deeper exploration of qualitative dimensions, to advance our understanding of burnout in this specific context and inform effective interventions for healthcare professionals.

## Data availability statement

The raw data supporting the conclusions of this article will be made available by the authors, without undue reservation.

## Ethics statement

Written informed consent was obtained from the individual(s) for the publication of any potentially identifiable images or data included in this article.

## Author contributions

SD: Conceptualization, Formal analysis, Methodology, Writing – original draft. PC: Conceptualization, Writing – original draft. OS: Conceptualization, Writing – review & editing. BK: Writing – review & editing. TS: Data curation, Formal analysis, Methodology, Writing – review & editing. ŠV: Writing – review & editing. JG: Supervision, Writing – review & editing. AS: Supervision, Writing – review & editing.
